# Porous Membranes Based on Polypropylene-Ethylene Copolymers. Influence of Temperature on Extrusion, Annealing and Uniaxial Strain Stages

**DOI:** 10.3390/polym10080854

**Published:** 2018-08-02

**Authors:** Pilar Castejón, David Arencón, Marcelo Antunes, Vera Realinho, José Ignacio Velasco, Antonio B. Martínez

**Affiliations:** Centre Català del Plàstic, Universitat Politècnica de Catalunya., C/Colom 114, 08222 Terrassa, Spain; pilar.castejon@upc.edu (P.C.); marcelo.antunes@upc.edu (M.A.); vera.realinho@upc.edu (V.R.); jose.ignacio.velasco@upc.edu (J.I.V.); antonio.martinez@upc.edu (A.B.M.)

**Keywords:** porous membranes, polypropylene-ethylene, extrusion

## Abstract

In this study, block and random copolymers of polypropylene–ethylene are selected to prepare porous membranes through the melt extrusion-annealing-uniaxial stretching technique (MEAUS), at a constant draw ratio. In some cases, these copolymers were blended with a homopolymer grade. The variation of temperature in the stages of extrusion, annealing and uniaxial strain was analysed. Several characterisation techniques were employed to study this influence. The crystalline orientation was analysed by polarised infrared spectroscopy (FT-IR), and crystalline features were studied by differential scanning calorimetry (DSC). The thermal stability of the membranes was checked by thermogravimetric analysis (TGA). Tensile tests were performed to ascertain the stiffness and ductility of the produced samples. The results were correlated with the porous morphology, global porosity, and permeability to air. A close relationship was found between crystalline characteristics, porous morphology and the trends registered. An improved pore distribution along the membrane surface was found when copolymers were employed.

## 1. Introduction

Polymeric microporous membranes are conventionally produced by two main techniques, one based on phase inversion process and the other is via melt processing extrusion, where a semi-crystalline polymer film with a specific structure is uniaxial stretched in order to create the porous morphology. This stretching method is commonly called MEAUS (melt extrusion-annealing-uniaxial stretching) and consists of three consecutive steps: (a) Melt extrusion of a precursor film with a row-nucleated lamellar structure; (b) annealing of the film to increase the lamellar thickness and remove irregularities; and (c) stretching of the annealed film at room and high temperature to create pores and enlarge their size [1,2,3,4,5,6,7,8,9,10,11,12,13,14,15,16,17,18,19,20,21]. Finally, the membranes should be heat-set to prevent shrinkage and to maintain the mean pore size constant [3,21].

Semi-crystalline homopolymers as polypropylene (PP) or polyethylene (PE) have been commonly employed to produce microporous membranes via MEAUS methodology. The use of this stretching process also facilitates the incorporation of mineral fillers to improve membrane performance [22,23,24,25]. Due to properties such as chemical and mechanical resistance, these polyolefins are used in a broad range of separation processes such as lithium batteries or gas/water filtration [26,27,28,29]. Overall, porosity and permeability properties are dependent on various factors. For example, control of molecular weight and polymer architecture to create a suitable lamellar structure, influence of different annealing time and applied temperature for precursor films, and the correct choice of stretching conditions, such as strain rate, stretch ratio, and strain temperature during the cold and hot uniaxial stretching steps.

Several studies have been reported to explain the effect of molecular weight, molecular weight distribution, and polymer architecture to create the row-nucleated structure required for the MEAUS methodology [2,3,4,5,6,7,8]. Sadeghi et al. [4] used five different polypropylene resins with distinct molecular weights. The results revealed that the main influence of this parameter on the orientation and lamellae connectivity. Smaller molecular weight resins reduce the crystalline and amorphous phase orientation, which leads to a decrease in the pore interconnection and membrane permeability. These authors [30] also examined the effect of stretching temperature and addition of polypropylene random copolymer on the properties of films prepared by two linear PP resins having different molecular weights. They concluded that these variables do not have a very strong effect on the machine direction orientation (MDO). However, higher drawing temperature resulted in the formation of fibrillar and lamellar crystalline structures, and the ethylene presence interfered with both fibril and lamellae crystallisation. Processing parameters during the melt extrusion process, such as the draw ratio and air-cooling applied on precursor films were also investigated [9]. In order to obtain a suitable row nucleated structure with higher orientation, an increment on draw ratio until an optimum value and a rapid air-cooling right at the exit of the extruder die must be applied.

Saffar et al. [18] investigated the influence of annealing process on membranes performance by applying different annealing time and temperature in polypropylene precursor films. They determined that a minimum temperature of 135 °C and 10 min were enough to increase lamellar thickness and improve lamellar morphology. Many studies have concluded that membrane characteristics as porosity, pore density and size, and permeability improve in films annealed before the stretching steps [13,14,15,16,17,18]. During stretching stages at room and high temperatures, when the porous morphology is achieved, the secondary crystalline lamellae created during the previous annealing step convert to the connecting bridges [2,3,4,5,6,7,8,9,10]. This is a new structure produced during lamellar separation, when some tie chains crystallise and separate the neighbouring pores. Influence of strain rate, stretch ratio, and stretch hot temperature were investigated by Saffar et al. [10]. To obtain greater permeability and uniform pores with higher size and interconnectivity, membranes may be produced at lower strain rate and a high hot temperature to increase flexibility of the amorphous phase. The authors also concluded that for annealed precursor films, an optimum strain percentage during stretching at room temperature, below the strain corresponding to the second yield point observed in stress-strain curves must not be exceeded in order to avoid lamellae fragmentation. The presence of this second yield point in the tensile curves was due to the deformation of the new recrystallised structure formed during the annealing process.

In a previous study [24], carried out by our research group, we evaluated the influence of applying various strain rates and stretch ratios at the same temperature during cold and hot stretching steps, on films prepared by filled and non-filled polypropylene resins. Membranes stretched at room temperature at medium strain rate and at an extent percentage close to 35% showed better permeability and porosity values. The same results are observed when membranes are produced at slower strain rate and higher strain extent at high temperature—when mobility of polymer chains increases and larger pore size can be obtained after greater lamellae separation.

In this study, different blends of a suitable linear polypropylene used from a previous work [24] with a block and random copolymers of polypropylene-ethylene were prepared. The influence of polymeric composition on membranes prepared through the MEAUS technique was evaluated. Blends with these copolymers were mainly prepared with the aim of producing homogenous porous membranes, increasing the pore size and pore size distribution. Samples from neat resins and their blends were produced at a constant draw ratio with different extrusion temperatures. Subsequently, during annealing and stretching steps, different temperatures were used to study the influence of this variable in the development of porous morphology; using (in some cases) extreme values of temperature (near the melting temperature). Changes produced on membrane structure were analysed by several characterization techniques. A detailed investigation trying to find a close relationship between crystalline features, porous morphology, permeability, and thermal and mechanical stability was performed.

## 2. Materials and Methods

### 2.1. Sample Compounding

Three commercial grades of homopolymer polypropylene and copolymer polypropylene–ethylene, with different molecular weight and polymer configuration, were kindly supplied by Repsol S.A (Madrid, Spain). The main features for the materials selected and the nomenclature used in the article are shown in Table 1.

In order to provide the same thermomechanical history to all the samples, neat resins and several compounds of these materials were prepared using a twin-screw extruder (Collin ZK-35, *L*/*D* = 36), with a temperature profile from hopper to die of 150–250 °C, and a screw speed of 60 rpm, and a circular die of diameter 3 mm. As a result, eight samples were obtained for this study and specifically referred to as H and B (100 wt %) H-5B (95–5 wt %), H-10B (90–10 wt %), H-20B (80–20 wt %), H-5R (95–5 wt %), H-10R (90–10 wt %), and H-20R (80–20 wt %).

### 2.2. Membrane Preparation

#### 2.2.1. Extrusion

**Figure 1:**
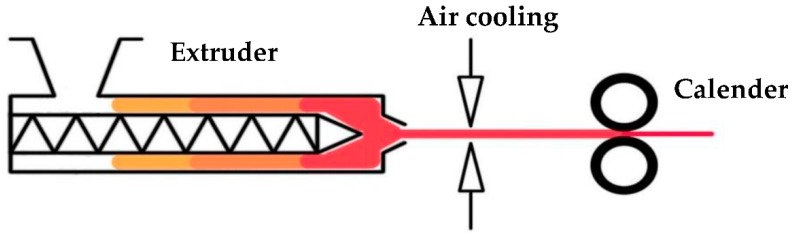
Scheme of precursor films manufacturing process.

The precursor films are mono-oriented films with a row-lamellar structure produced during the melt-extrusion process (first step of the MEAUS technique), which can be converted to the corresponding porous membranes. These precursor films were produced using a twin-screw extruder (Collin ZK-35, *L*/*D* = 36, Dr Collin GmbH, Ebersberg, Germany) equipped with a slit die 25 mm-width, and 1.9 mm thickness. To study the temperature influence on the properties of materials during this stage, samples were produced at two different temperature profiles from hopper to die (150–250 °C and 150–270 °C). On the other hand, high draw ratio and a fast cooling at the exit of the extrusion die were applied using a calendar system with variable speed, and slit-open air knives that supply air at a pressure of 2 bar to both sides of the mono-oriented precursor films (Figure 1). The draw ratio applied (70 in all samples) was determined by dividing the linear calendaring rolling speed into the linear speed of the molten material at the exit of the extruder die.

#### 2.2.2. Annealing and Uniaxial Stretching

From the precursor film with a ca. 30 μm thickness, rectangular samples were cut out with a width and length of 60 and 100 mm, respectively, to be annealed without extension at four different temperatures (140, 150, 160 and 170 °C) in an air-circulating oven during 15 min.

Then, a universal testing machine equipped with a climatic chamber was employed to perform the uniaxial stretching of the annealed precursor films in two different steps. The first one was at room temperature (cold stage) at a drawing speed of 50 mm/min up to 35% percent elongation. In the second step (hot stage), samples were stretched at three different temperatures (140, 150, and 160 °C) at 10 mm/min strain rate up to 230% of the initial length. The uniaxial strain direction was parallel to the drawing direction of the precursor film during the extrusion (MDO—machine direction orientation). Once the test was finished and before relieving the films from the tensile grips, the membranes were kept at the same hot temperature stretching for 90 s to stabilize the porous morphology.

### 2.3. Membrane Characterization

The crystalline characteristics were analysed by Fourier-Transmission Infrared spectra using a Perkin Elmer 1000 FTIR (Perkin Elmer, Waltham, MA, USA) infrared spectrometer, with a spectral resolution of 1 cm^−1^ and polarized beam. The crystalline orientation was determined for samples non-annealed and annealed at different temperatures based on the difference of the absorption in the two orthogonal directions, parallel (*A*_0_) and perpendicular (*A*_90_) to the reference axis MD (extrusion machine direction). For the crystalline phase orientation (*F*_c_), the data was obtained from the range of 4000 to 600 cm^−1^ at the wavelength of 998 cm^−1^. The Herman orientation function was calculated according to Equation (1):

*F*_c_ = (*D* − 1)/(*D* + 2) (1)

where dichroic ratio (*D*) is the ratio of both absorbencies, parallel and perpendicular (*A*_0_/*A*_90_).

The microporous membranes surfaces were observed using a scanning electron microscopy (SEM) with a JEOL JSM-7001F microscope operated at 2 kV voltage (JEOL, Akishima, Japan), and the samples were coated with a thin layer of gold in argon atmosphere employing a BAL-TEC SCD005 Sputter Coater (BAL-TEC, Balzers, Liechtenstein). From several SEM micrographs done for each sample, the membrane surface morphology was characterised and the number of pores, pores area and average pore size were determined using an image analysis program (Buehler Omnimet, version, Omnimet Advanced, Buehler, Lake Bluff, IL, USA, 2015). For calculation purposes, a circular-like porous geometry was assumed. The average pore density of the membranes was calculated with consideration for the number of pores divided by the membrane surface area.

Air permeability of porous membranes was measured with a Gurley densometer (Lorentzen & Wettre, ABB, Zurich, Switzerland) according to the international standard ISO 5636-5. The Gurley value was defined as the time (*t*) for a settled volume (100 mL) of air passing through the sample with a fixed area (0.79 cm^2^) under a pressure of 0.02 MPa. Normally, a longer time corresponds to a low air permeability and a long tortuous path for air transportation through the pores. The Gurley permeability value was obtained using the following equation:

Gurley permeability = 135.5/*t* (2)

Porosity estimation of all the membranes was predicted by imbibition of the membranes into the water during 24 h using the grain volume method. Firstly, the dry weight of the samples (*W*_dry_) and the saturated weight (*W*_sat_) after imbition were determined. The difference of weight is divided by the density of water (ρ_water_) to find the pore volume (*V*_p_) of samples as follows:

*V*_p_ = (*W*_sat_ − *W*_dry_)/ρ_water_ (3)

Hence, using the density of the samples (ρ_objective_) and applying Equation (4), it is possible to find the *V*_g_ value:

*V*_g_ = *W*_dry_/ρ_objective_ (4)

The percentage of porosity (Φ) was obtained by dividing the *V*_p_ by *V*_b_ as given in Equation (5):

Φ = (*V*_p_/*V*_b_) × 100 (5)

where *V*_b_ is the sum of *V*_p_ and *V*_g_.

In order to analyse the thermal properties and amorphous-crystalline phase transformations, the differential scanning calorimetry (DSC Q2000, TA instruments, New Castle, DE, USA) technique was employed. Calibration of the instrument was done by using standard samples of indium and lead. The sample mass was ca. 5–7 mg. Samples were heated from 30 to 200 °C at a heating rate of 10 °C/min. Samples percent crystallinity was calculated according to Equation (6):

*X*_m_ = (∆*H*_m_/∆*H*_m_^0^) × 100 (6)

where ∆*H*_m_ is the melting enthalpy measured in the resulting thermograms by integrating the area under the peaks (J/g) and ∆*H*_m_^0^ is the theoretical enthalpy of PP 100% crystalline (207.1 J/g).

Thermal stability of the membranes was evaluated using a TGA/DSC 1 Mettler Toledo Star System Mettler Toledo, Columbus, OH, USA. Thermogravimetric analyses were performed heating samples from 40 to 700 °C at a heating rate of 10 °C/min, and three different analyses were done for each membrane (sample weight of ca. 8.0 mg) using a constant running flow of air (60 mL/min).

The mechanical properties along MD (machine direction) were tested on the annealed precursor films using a universal tensile machine SUN2500, (Galdabini, Cardano al Campo (VA), Italy) with 1 kN load-cell equipped with a videoextensometer (MINSTRON OS-65D Minstron, Taipei, Taiwan). Tensile specimens were stretched at room temperature at 50 mm/min, the strain rate applied during the first uniaxial stretching step. With the resulting engineering stress-strain curves, the influence of polymer composition and the annealing temperature on membrane performance were analysed.

## 3. Results and Discussion

This section will be divided into four main headings: (Section 3.1) Influence of extrusion and annealing temperature on neat resins; (Section 3.2) influence of annealing temperature on polymer blends; (Section 3.3) the effect of the hot temperature applied during the uniaxial stretching step; and finally (Section 3.4) the influence of polymer composition and annealing temperature on mechanical and thermal stability of membranes.

### 3.1. Influence of Extrusion and Annealing Temperature on Neat Resins

**Figure 2:**
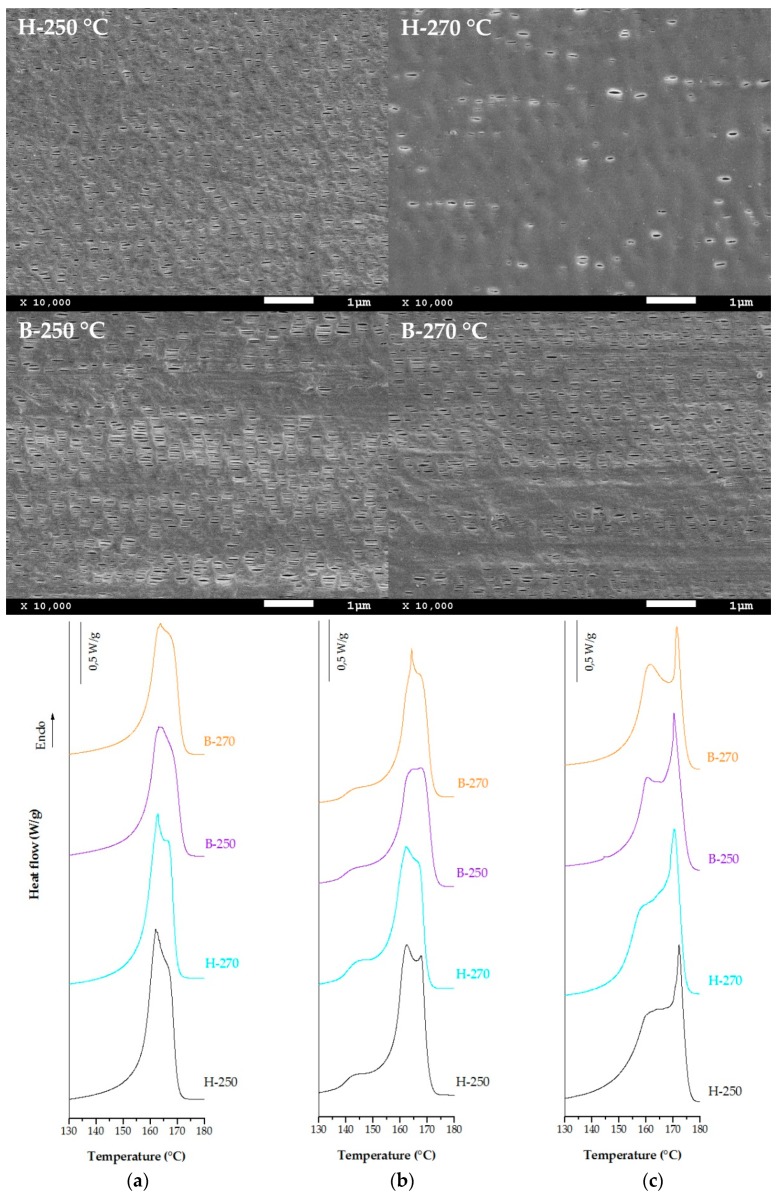
Scanning electron microscopy (SEM) micrographs and differential scanning calorimetry (DSC) thermograms of samples H and B extruded at different temperatures (250 and 270 °C), (**a**) precursor films, (**b**) annealed precursor films at 140 °C, (**c**) membranes.

In the first step of MEAUS technique is possible to control some of the properties of precursor films, which will become membranes during subsequent stages. Variables of melt extrusion process as draw ratio and air-cooling applied to fix the crystalline structure have already been studied [6,9]. For this investigation, a homopolymer resin of polypropylene and a heterophasic and random ethylene-polypropylene copolymers were selected to investigate the influence of processing temperature on these materials. The temperature profile from hopper to die for all the resins was from 180 to 250 °C, and from 180 to 270 °C. Figure 2 presents SEM micrographs of membranes produced with the homopolymer and block copolymer (annealing at 140 °C), and DSC thermograms for precursor films before and after the annealing process, and after the uniaxial stretching step. The neat PP random copolymer (100 wt %) does not form porous membranes and for this reason the data have not been included in this study.

Thermal analyses were conducted to evaluate possible microstructural changes during the annealing and uniaxial stretching steps. From the DSC thermograms shown in Figure 2a,b, it can be observed the presence of a shoulder at lower temperatures from the main melting peak related to the annealing of the precursor films. During this process, a new secondary crystalline structure was formed between the amorphous and the main crystal lamellae. High annealing temperatures promote an increment on molecular mobility and structural rearrangement increasing lamellar thickness and reducing irregularities [15,16,17,18]. From the DSC curves shown in Figure 2c, corresponding to membranes produced after annealing, this left shoulder disappears, and the total area is wider, which is attributed to the presence of a broad range of crystalline structures with different lamellar thicknesses. Moreover, the melting temperature increases, and a more intense right peak is observed due to the molecular orientation induced by stretching. During this uniaxial stretching step, pores were formed and pore size increases with greater lamellae separation. This right peak has been related to the stable crystalline form achieved in this step, where some tie chains are stretched and promote the crystallization of the so-called “interconnected bridges” that join main crystal lamellae and divide the pores [2,4,10].

For the neat resins extruded at different temperatures, the endotherms curves were quite similar, only for the block copolymer the right shoulder is more pronounced due to presence of long and thicker interconnected bridges as the pore size is larger in these samples (Table 2). The main differences are found on SEM micrographs taken from the surface of membranes and from values collected in Table 2. In all samples, when we increase the annealing temperature of precursor films, the orientation factor determined by polarised infrared spectroscopy (FT-IR) is increased. This phenomenon is due to an increment of the molecular rearrangement kinetics into a more dense and stable secondary form [11,18]. Homopolymer and block copolymer extruded at the same draw ratio, but at temperature above 250 °C promote the formation of a row-nucleated structure with a lower orientation factor, which reduces the porosity and permeability of membranes. It is important to mention that for the polypropylene random copolymer, which has higher viscosity, the optimum profile temperature for melt extrusion process was above 250 °C. For this reason, blends with this resin were prepared at the profile temperature from 180 to 270 °C.

Comparing the micrographs for samples H and B, the pore density value is quite similar, but it can be noticed that larger pore size was obtained when membranes were produced with the heterophasic polypropylene. The ethylene content present on the resin showed a melting temperature at 120 °C in comparison with the 165 °C associated with the polypropylene. Therefore, during the uniaxial stretching at high temperature, the ethylene regions distributed into de PP matrix are above their melting point, leading to a greater lamellae separation and an increment in both, pore size and air permeability values. Nevertheless, the ethylene content of the samples interferes with lamellae crystallization [30] and promotes the appearance of non-uniform areas in membranes prepared with the heterophasic polypropylene compared to the homopolymer, as can be seen in the SEM images. For this reason, this study aims to combine both effects by adding different percentages of PP copolymer in the homopolymer resin to achieve homogeneous membranes with greater permeability by increasing the pore size. Finally, for both neat resins the optimum annealing seems to be close to 160 °C. This temperature value, near the melting point of polypropylene, increases the air permeability with increasing the density, area and size of pores. This behaviour is a consequence of the softening of the crystalline region and the increment of flexibility in the amorphous phase at high temperatures.

### 3.2. Influence of Annealing Temperature on Polymer Blends

**Figure 3:**
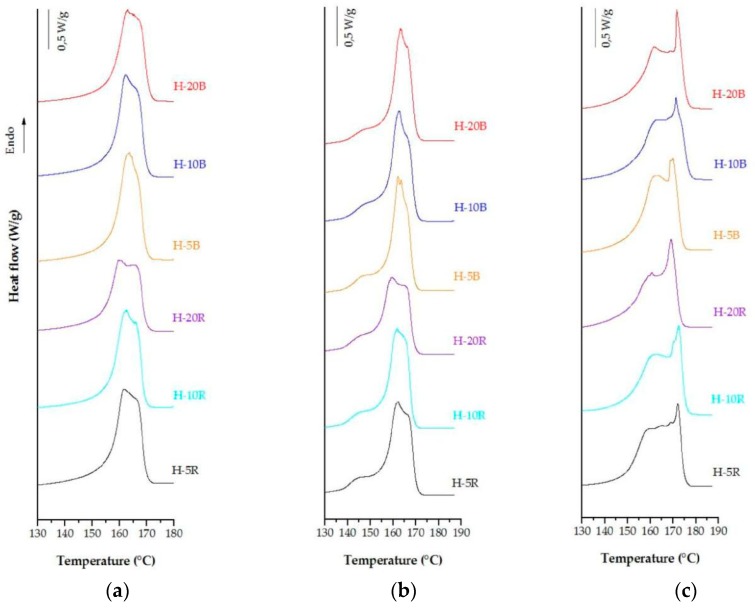
DSC thermograms of polymer blends prepared with the polypropylene (PP) random and block copolymers, (**a**) precursor films, (**b**) annealed precursor films at 140 °C, and (**c**) membranes.

Polymer blends with PP block and random copolymers were prepared at three different percentages in order to evaluate the effect of blending ratio and annealing temperature. Figure 3 illustrates the DSC curves of samples annealed at 140 °C, and uniaxial stretched in two different steps, one at room temperature and the other at 140 °C.

Endotherm curves for blends with the random copolymer were less pronounced and wider as the content of the mixture was increased, in comparison with the curves related to the blends with the heterophasic polypropylene. This behaviour is attributed to a broad range of crystals with different lamellar thickness [31]. As the random content in the blend increases (H-20R), a decrease in the melting point is also observed. This is expected due to some typical characteristics of random copolymers, as their lower crystallinity and polymer architecture [27,32,33,34,35]. The analysis of experimental data (Table 3 and Table 4) and the SEM examinations revealed that membranes differing on blending ratio have an optimum value close to 10 percent for the random copolymer, and 20 percent for the heterophasic PP—where higher pore density and a pore distribution fairly homogeneous were achieved. On the basis of the data obtained, the effect of annealing process on membrane performance for the sample H-10R and H-20B was investigated.

**Figure 4:**
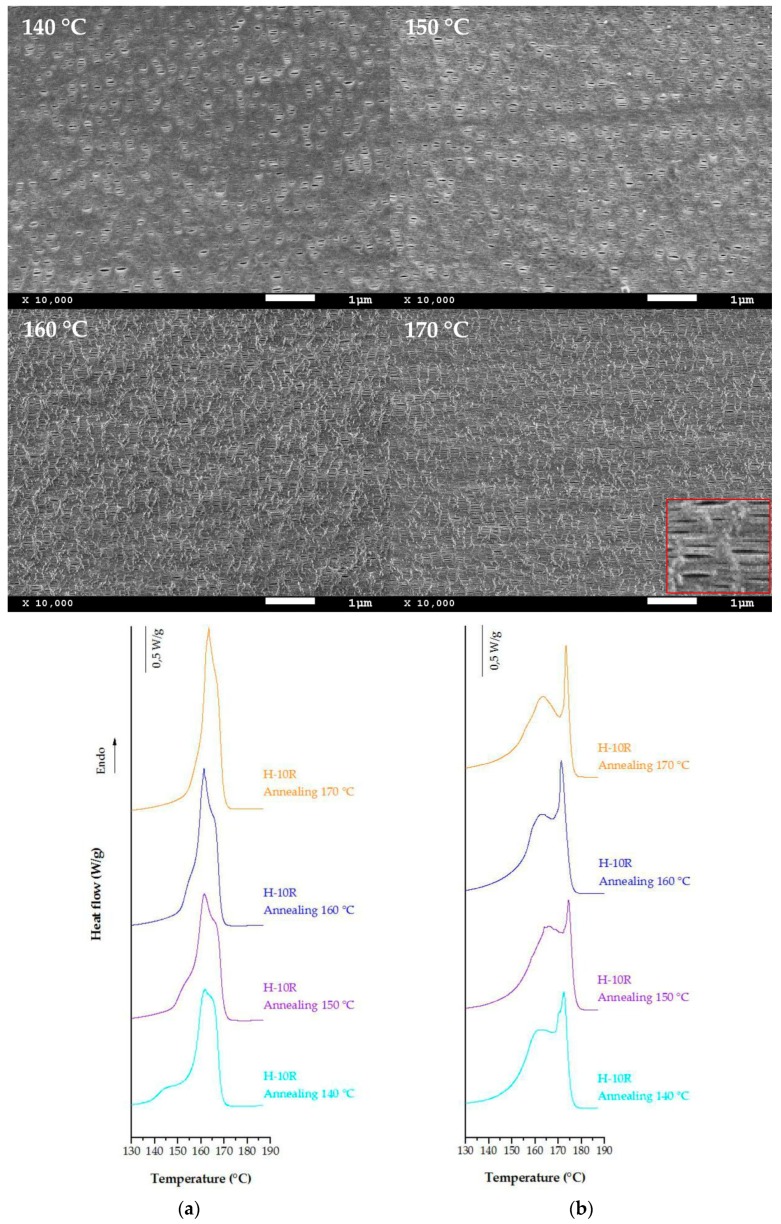
SEM micrographs and DSC thermograms of sample H-10R annealed at different temperatures (**a**) annealed precursor films, and (**b**) membranes (second stretching step at 140 °C).

The SEM images showing the surface morphology of membranes that were produced with 10 percent random copolymer and annealed at four different temperatures are represented in Figure 4. With a blend ratio above this 10 percent, a non-optimum row-nucleated structure is formed; therefore, air permeability and morphological membrane properties were not improved (Table 3). High annealing temperature causes an increment in the number of pores, as well as on the orientation factor determined by FT-IR. Comparing the data collected in Table 3, it was observed that membranes produced until a 10 percent of blend having higher porosity and air permeability values were obtained for the precursor films annealed at 160 °C. At this temperature, chain rearrangements promote an increment of lamellar thickness with increasing the degree of crystallinity. This phenomenon and the influence of the ethylene content during the hot uniaxial stretching step produce higher pore size and better interconnectivity between the pores. Endotherms curves obtained from precursor films and membranes annealed at these temperatures are also showed in Figure 4.

DSC thermograms above 140 °C display significant changes in the shape of melting curves. Shoulder at lower temperatures related to the annealing process [14,15,16,17,18] and also associated with the melting of different crystal populations disappears progressively as the annealing temperature increases. It seems that the increment on molecular mobility at higher temperatures promotes the formation of a more dense and stable crystalline form [31]. Membrane thermograms display similar endothermic curves for annealing temperatures above 150 °C. Two types of lamellae distribution after uniaxial stretching appear, and peaks at high temperatures related to the interconnected bridges formed between main crystal lamellae were more pronounced for samples annealed at 160 °C and 170 °C. Although the annealing temperature of 170 °C increases the precursor film orientation and enhances pore density, a reduction in the pore size reduces permeability properties. This could be explained as a deterioration of crystalline lamellar structure due to a partial melting of some areas, which produces a loss in membrane surface homogeneity and pore interconnectivity.

**Figure 5:**
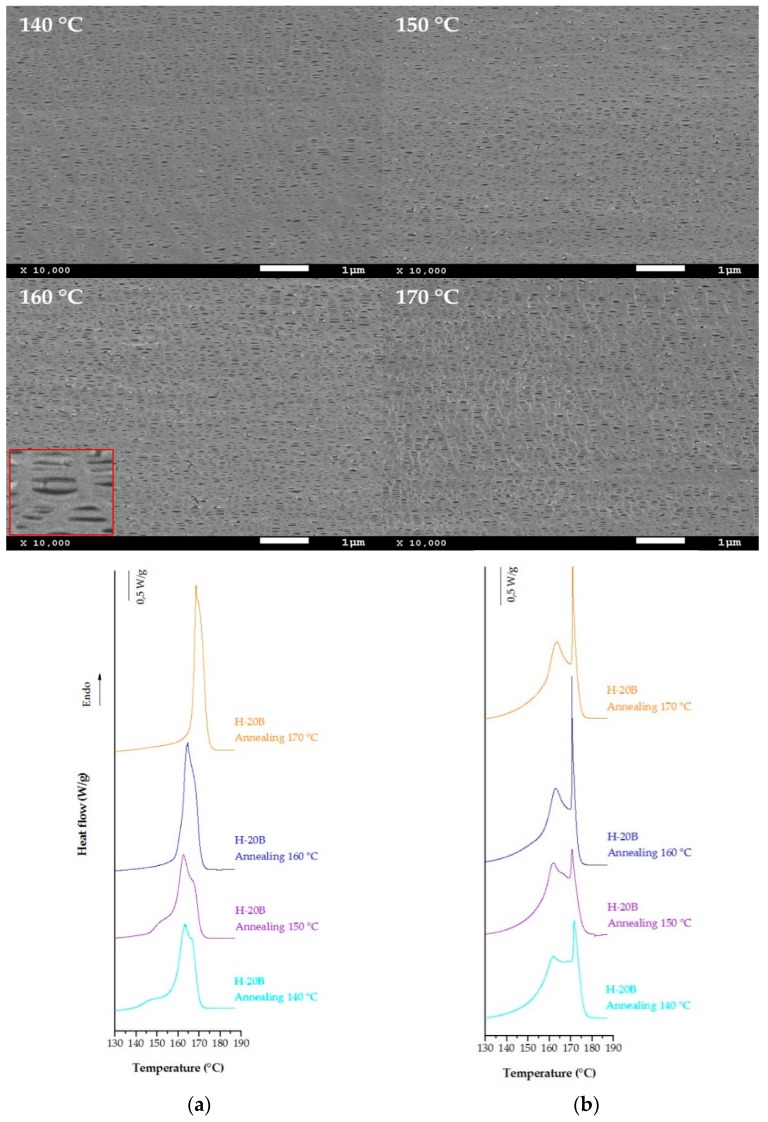
SEM micrographs and DSC thermograms of sample H-20B annealed at different temperatures (**a**) annealed precursor films, and (**b**) membranes (second stretching step at 140 °C).

Blends of the homopolymer and the heterophasic polypropylene copolymer at three different percentages were prepared to obtain larger air permeability values and homogeneous membranes. The SEM micrographs and DSC thermograms shown in Figure 5 are for the sample with 20 percent of blend. This increment on blending ratio causes a significant increase of membrane permeability due to a presence of a larger number of pores. DSC thermograms of precursor films annealed at a temperature above 150 °C showed similar results for both, random and block copolymers, where the higher temperatures seems not to form the secondary crystal lamellae between amorphous phase and main crystal lamellae. The crystallinity is similar in all cases, in spite of the difference observed in the peak corresponding to the recrystallized interconnected bridges formed during the hot stretching step. This narrower melting peak becomes more pronounced for membranes which precursor films were annealed at 160 °C (when a more stable crystalline structure improved membrane performance by increasing pore size and number of pores). When the annealing temperature was further increased to 170 °C, the melting of the crystalline phase induces a heterogeneous pore morphology that would decrease the air permeability, pore density and size, and the interconnectivity of pores through the membrane.

### 3.3. Influence of Temperature during Hot Stretching Step

**Figure 6:**
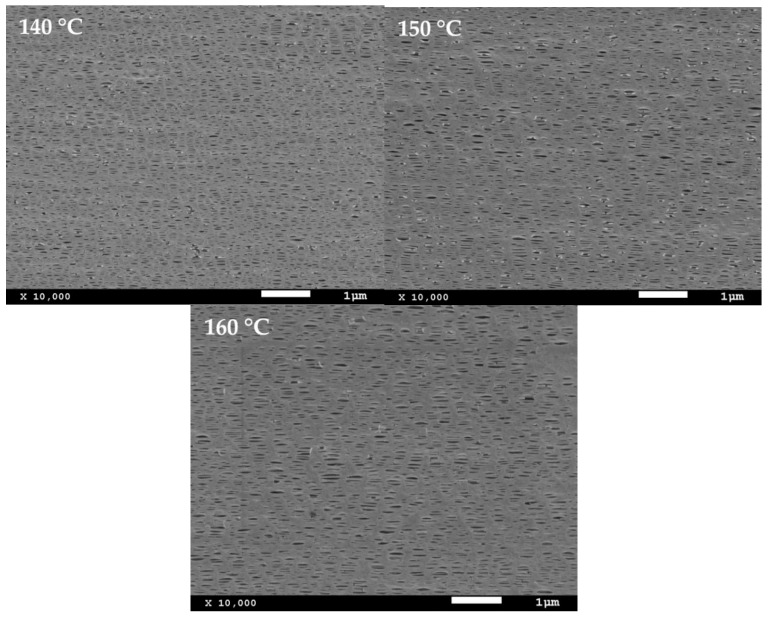
SEM micrographs of sample H-20B. Precursor films annealed at 160 °C and stretched at three different temperatures during the second uniaxial stretching step.

The influence of hot stretching temperature on microporous membranes obtained from blends with the heterophasic and random PP copolymers was also investigated. Three different temperatures were applied trying to enhance the pore size and air permeability. All the samples showed similar behaviour; therefore, only the results corresponding to the sample H-20B are presented in Figure 6 and Table 5. These results showed that increasing temperature above 140 °C during the second uniaxial stretching step produces higher pore size with longer recrystallized connecting bridges between lamellae. At these high temperatures, an increment of flexibility of tie chains located in the amorphous phase [10], and the melting of ethylene content present on PP matrix allow greater lamellar separation to enhance the pore size. The size increment was also related to a collapse occurred between neighbouring pores, when some interconnected bridges break with high elongation values promoting higher average pore size at the expense of reducing pore density [24]. However, temperatures so close to the melting point of the material cause local melting of the plastic decreasing the membrane performance.

### 3.4. Effect of Polymer Composition and Annealing Temperature on Mechanical and Thermal Stability

**Figure 7:**
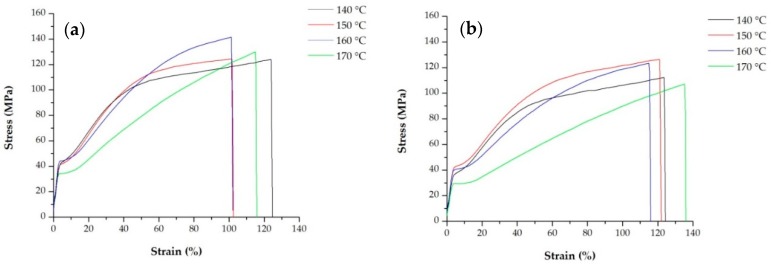
Stress-strain curves of samples (**a**) H-20B and (**b**) H-10R. Precursor films annealed at four different temperatures.

In Table 6 and Table 7 are presented the average value of Young’ modulus, and the first yield stress point for each sample. The curves presented in Figure 7 show the response to applied stress of two types of precursor films annealed at four different temperatures.

For all samples, during the stretching of the film, a stress-whitening phenomenon was observed [36,37]. After yielding, the tensile curves presented an increase in the nominal stress with the beginning of the strain hardening. The stress applied involved a pre-orientation of amorphous tie chains prior to crystal chain unfolding, which can be related to the first yield point [31]. Annealing the samples at 150 and 160 °C increased the yield stress and elastic modulus due to an increment of sample crystallinity. On the other hand, the strain hardening or second yield point related to the deformation of the secondary crystalline lamellae (formed during annealing process) was less noticeable for samples annealed at temperatures above 150 °C. These results are in accordance with the endothermic curves reported in Figure 4 and Figure 5, where for samples annealed above this temperature the shoulder on the left side of the main melting peak, associated with the secondary new crystalline region disappears to produce a more dense and stable crystalline structure. The pronounced strain hardening after yielding can be promoted by homogeneous crystal slip that makes the plastic deformation more difficult [18,31]. For this reason, when an optimum percentage near to the strain of 35% is achieved [10,24], the second uniaxial stretching step at high temperatures begins and thermal effects counteracting the strain hardening. Thus, the total pore size increases with greater lamellae separation. Annealing temperature above 160 °C significantly reduces the mechanical properties of membranes due to changes produced on the crystalline structure.

**Figure 8:**
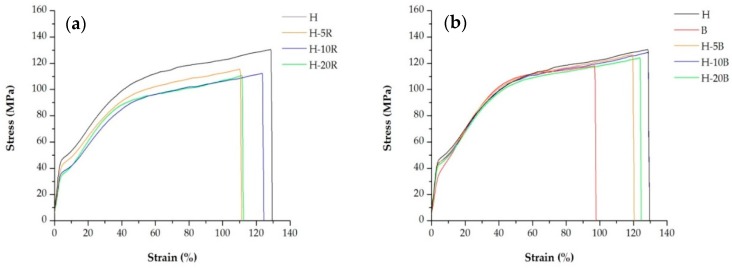
Stress-strain curves of precursor films annealed at 140 °C, for the PP homopolymer and its blends with the (**a**) random and (**b**) block copolymers.

In Figure 8, the engineering stress-strain curves show the response of precursor films annealed at the same temperature, as the blending ratio with both, block and random copolymers increases. Decreased mechanical stiffness and yield stress at high random and block copolymer content were observed. The heterophasic PP copolymer showed a diffusive yielding point followed by a stronger strain-hardening compared with the homopolymer, whereas that for their blends intermediate values have been obtained. These results showed that with less stiff resins and until the optimum value in the uniaxial stretching step at room temperature [10,24], less force is required for the lamellae separation that forms pores in membranes. Increased ductility was expected for membranes prepared from these copolymers, but the results in the maximum strain achieved were not as expected. This could be attributed to the low thickness of the samples, where any type of irregularity, chain entanglement or other defects could promote material failure.

**Figure 9:**
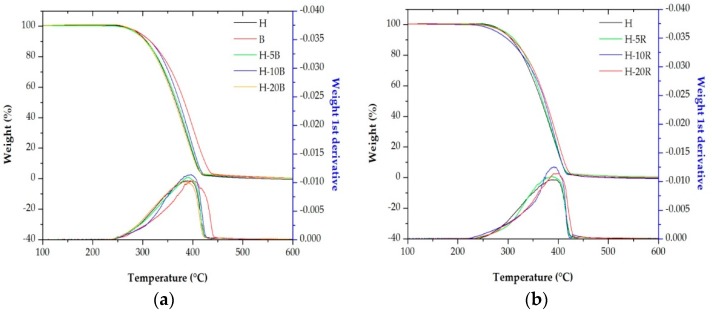
TGA and DTG curves of membranes annealed and stretched at the same temperature for the PP homopolymer and its blends with the (**a**) block and (**b**) random copolymers.

**Figure 10:**
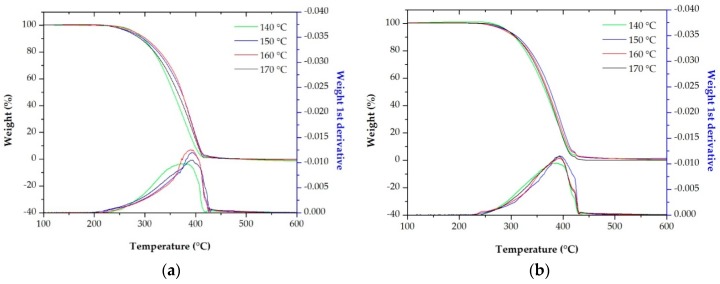
Thermogravimetric analysis (TGA) and its respective first derivative (DTG) curves of samples (**a**) H-10R and (**b**) H-20B. Precursor films annealed at different temperatures and membranes prepared under the same stretching conditions.

The thermogravimetric analysis (TGA) and its respective first derivative (DTG) for neat polymers and their blends were performed to investigate the effect of blending ratio, and the influence of annealing temperature on the thermal stability of membranes. From these curves (Figure 9 and Figure 10), values collected in Table 8 show the temperature for a loss of mass of 10 wt % (*T*_0.1_), 50 wt % (*T*_0.5_), relative loss of mass at 400 and 600°C, and the maximum decomposition rate temperature (*T*_max_). Thermal degradation mechanism of polypropylene includes different reactions, such as initiation, propagation and termination. All TGA curves were characterized by a one-single stage of decomposition, where the only decomposition variable considered was the loss of mass related to evolution of volatile material [35,38,39,40].

Pure PP homopolymer resin begins to volatize at less temperature than pure heterophasic PP copolymer, as can be seen in Figure 9a. The membrane thermal stability was slightly increased up to about 10 blend percent of block copolymer in the homopolymer resin. It is deduced from Figure 9b that something similar happened when the content of random copolymer was increased up to about 20 percent of blend. Higher percentages have resulted in a thermal resistance decrease, due to the tendency to immiscibility increased with higher PP copolymer content [27,34,35].

Other factor that had to be involved in the analysis was the influence of temperature annealing on the thermal stability of membranes. Thermogravimetric analyses were employed for membranes with higher permeability values produced using the random (H-10R) and block (H-20B) copolymers to study this effect. The TGA curves and the first derivate mass loss (DTG) presented in Figure 10 reported the influence of this parameter. Higher annealing temperatures enhanced the thermal stability of the samples for blends of both, block and random copolymers as seen from the results collected in Table 9. As noted by other authors [11,12,13,14,15,16,17,18], annealing leads to improvements of membrane properties by thickening the lamellae and eliminating internal irregularities. As pointed out in the previous sections, one of the changes produced in the crystalline structure during annealing is an increase in crystallinity. Thermal stability is enhanced when a reduction in fraction of amorphous phase is achieved at higher annealing temperatures, due to a decrease in the air absorbed at molecular level, which has a negative influence in the membrane thermal resistance [39].

## 4. Conclusions

The polymer composition and the temperature variation in the main stages of the MEAUS technique, such as the melt extrusion, annealing and the second stage of uniaxial stretching have been analysed in this work.

Neat polypropylene random copolymer has resulted in a not a suitable material to produce porous polymeric membranes by the MEAUS technique, due to its low degree of crystallinity. Nevertheless, homogeneous porous membranes with high air permeability were obtained when the random copolymer percentage in PP homopolymer blend was up to about 10%. This is mainly due to the less stiffness and the ethylene content. The microporous membranes with the highest air permeability and the most homogeneous pore structure were obtained from blends up to a 20 percent of the heterophasic copolymer in the PP homopolymer. The ethylene content and polymer configuration of the block copolymer promote higher lamellar separation increasing the pore size.

Precursor films extruded at high temperatures showed a lower oriented crystalline structure decreasing the membranes performance. Regarding to the annealing process, an increment in temperature gives rise to a greater molecular orientation and a rearrangement of polymer chains in both, the crystalline and amorphous phases, which leads to an increase in crystallinity. The samples annealed just up to 160 °C showed the highest pore structure homogeneity and permeability values (Table 2, Table 3 and Table 4). Above this temperature, the melting of some crystals reduces pore density, pore size, and interconnectivity of pores.

The rise in temperature during the second uniaxial stretching step increases the molecular mobility, obtaining a higher pore size. However, uniaxial stretching above 140 °C produces non-homogeneous samples decreasing the membrane performance.

Lower stiffness for blends with both, block and random copolymers, and less noticeable strain hardening in samples annealed at temperatures above 150 °C may promote greater lamellae separation. The membranes prepared with the block and random copolymers until an optimum value and under high annealing temperatures showed an improved thermal stability.

## Figures and Tables

**Table 1:** Commercial polypropylene and polypropylene–ethylene resins selected.

Commercial Tradename	Code	Polymer Configuration	MFI ^a^ (dg/min)
ISPLEN PP020 G3E	H	Homopolymer	0.9
ISPLEN PB110 H2E	B	Block Copolymer	0.3
ISPLEN PR230 C1E	R	Random Copolymer	1.6

^a^ Melt flow index measured according ISO 1133 (230 °C/2.16 kg).

**Table 2:** Characteristics of neat resins extruded and annealed at different temperatures in terms of orientation of precursor films, and membrane crystallinity, pore morphology, and air permeability.

Sample	Extrusion (°C)	Annealing (°C)	*F*_c_	*X*_m_ (%)	Pore Density (Pores/μm^2^)	Pores Area (%)	Average Pore Size (µm)	Porosity (%)	Gurley Permeability (µm/(Pa·s))
H	250	140	0.69	52.4	9.8	3.9	0.065	0.46	0.004
		150	0.72	54.5	8.3	3.0	0.061	0.48	0.004
		160	0.76	56.1	14.2	5.0	0.062	0.53	0.005
		170	0.79	55.8	13.9	4.9	0.063	0.49	0.004
H	270	140	0.59	53.3	1.2	0.7	0.072	0.41	0.003
		150	0.66	55.1	6.0	2.9	0.071	0.41	0.003
		160	0.70	55.8	12.5	5.4	0.070	0.44	0.003
		170	0.71	55.6	11.5	3.9	0.062	0.43	0.003
B	250	140	0.74	50.7	9.4	7.7	0.091	0.62	0.007
		150	0.76	48.5	11.9	8.6	0.091	0.78	0.018
		160	0.78	49.4	13.1	11.3	0.098	1.65	0.068
		170	0.79	47.4	10.9	6.4	0.086	1.29	0.020
B	270	140	0.69	49.1	10.0	6.1	0.076	0.55	0.005
		150	0.69	49.2	11.4	8.2	0.088	0.65	0.013
		160	0.76	49.9	12.2	8.3	0.083	0.95	0.026
		170	0.77	49.6	7.5	5.3	0.080	0.62	0.011

**Table 3:** Characteristics of membranes prepared with the PP random copolymer under the same stretching temperature (140 °C), differing on blending ratio and annealing temperature.

Sample	Annealing (°C)	*F*_c_	*X*_m_ (%)	Pore Density (Pores/μm^2^)	Pores Area (%)	Average Pore Size (µm)	Porosity (%)	Gurley Permeability (µm/(Pa·s))
H-5R	140	0.60	52.0	1.5	0.9	0.062	0.41	0.003
	150	0.66	57.3	10.4	3.3	0.059	0.46	0.003
	160	0.73	59.9	13.7	5.1	0.065	0.45	0.003
	170	0.73	53.7	13.8	4.5	0.058	0.49	0.004
H-10R	140	0.67	53.9	6.9	2.4	0.062	0.45	0.004
	150	0.68	58.2	11.2	3.1	0.066	0.55	0.008
	160	0.74	59.5	14.2	5.8	0.070	0.85	0.048
	170	0.76	56.1	13.9	5.1	0.069	0.75	0.039
H-20R	140	0.72	46.3	7.7	2.6	0.063	0.45	0.003
	150	0.75	55.6	6.0	2.2	0.066	0.55	0.006
	160	0.76	59.6	7.8	3.2	0.069	0.59	0.019
	170	0.77	51.3	7.6	2.7	0.063	0.56	0.013

**Table 4:** Characteristics of membranes prepared with the PP heterophasic copolymer under the same stretching temperature (140 °C), differing on blending ratio and annealing temperature.

Sample	Annealing (°C)	*F*_c_	*X*_m_ (%)	Pore Density (Pores/μm^2^)	Pores Area (%)	Average Pore Size (µm)	Porosity (%)	Gurley Permeability (µm/(Pa·s))
H-5B	140	0.60	50.7	16.9	8.7	0.071	0.73	0.010
	150	0.64	53.1	17.8	9.3	0.070	0.79	0.011
	160	0.69	54.6	20.6	9.9	0.073	0.85	0.011
	170	0.74	54.1	20.6	10.1	0.075	1.02	0.010
H-10B	140	0.68	49.1	24.3	8.4	0.070	1.02	0.044
	150	0.70	55.9	22.6	9.0	0.071	1.16	0.032
	160	0.74	56.1	25.8	9.1	0.068	1.85	0.160
	170	0.75	55.7	25.1	7.8	0.065	1.67	0.110
H-20B	140	0.68	49.5	26.2	8.8	0.075	1.69	0.132
	150	0.71	53.6	26.5	10.7	0.078	1.69	0.120
	160	0.74	53.2	27.8	11.6	0.079	1.92	0.176
	170	0.75	53.0	25.3	10.9	0.076	1.86	0.151

**Table 5:** Characteristics of membranes prepared at different temperatures during the second stretching step in terms of membrane crystallinity, pore morphology, and air permeability.

Sample	Annealing (°C)	Hot Stretching (°C)	*X*_m_ (%)	Pore Density (Pores/μm^2^)	Pores Area (%)	Average Pore Size (µm)	Porosity (%)	Gurley Permeability (µm/(Pa·s))
H-20B	160	140	53.2	27.8	11.6	0.079	1.92	0.176
		150	53.1	17.9	13.2	0.085	1.59	0.093
		160	54.0	12.7	10.1	0.089	1.01	0.081

**Table 6:** Results of tensile test along MD (extrusion machine direction) for precursor films of PP homopolymer and blends with the random and block copolymers.

Sample	Young’s Modulus (GPa)	First Yield Stress Point (MPa)
H	1.71 ± 0.05	47.8 ± 0.7
B	0.97 ± 0.04	37.9 ± 0.3
H-5B	1.69 ± 0.05	45.6 ± 0.5
H-10B	1.59 ± 0.05	45.5 ± 0.6
H-20B	1.49 ± 0.05	43.9 ± 0.3
H-5R	1.49 ± 0.03	44.6 ± 0.5
H-10R	1.20 ± 0.04	37.8 ± 0.5
H-20R	1.08 ± 0.05	35.9 ± 0.6

**Table 7:** Results of the tensile test along MD for precursor films of PP random and block copolymers after different annealing temperatures.

Sample	Annealing (°C)	Young’s Modulus (GPa)	First Yield Stress Point (MPa)
H-10R	140	1.20 ± 0.04	37.8 ± 0.5
	150	1.31 ± 0.02	43.0 ± 0.5
	160	1.31 ± 0.04	40.8 ± 0.5
	170	1.01 ± 0.05	27.9 ± 0.4
H-20B	140	1.49 ± 0.05	43.9 ± 0.3
	150	1.48 ± 0.04	42.9 ± 0.4
	160	1.49 ± 0.05	44.3 ± 0.6
	170	1.39 ± 0.05	33.9 ± 0.6

**Table 8:** Thermogravimetric results for membranes of PP homopolymer and blends with random and block copolymers.

Sample	*T*_0.1_ (°C)	*T*_0.5_ (°C)	Lost Mass 400 °C (%)	Lost Mass 600 °C (%)	*T*_max_ (°C)
H	307.9	366.5	83.6	100.0	389.7
B	315.7	383.7	66.3	99.7	400.4
H-5B	306.7	368.9	82.2	99.7	391.7
H-10B	315.5	373.8	78.9	99.7	396.9
H-20B	305.9	367.4	85.3	99.8	390.7
H-5R	313.4	368.9	82.9	99.6	388.8
H-10R	301.1	373.3	82.5	100.0	391.2
H-20R	309.9	375.7	76.4	99.9	399.6

**Table 9:** Thermogravimetric results for blends prepared with the PP random and heterophasic copolymers after four different annealing temperatures.

Sample	Annealing (°C)	*T*_0.1_ (°C)	*T*_0.5_ (°C)	Lost Mass 400 °C (%)	Lost Mass 600 °C (%)	*T*_max_ (°C)
**H-10R**	140	296.6	356.3	91.2	100.0	379.5
	150	295.7	370.8	81.7	100.0	393.2
	160	301.1	373.3	82.5	100.0	391.2
	170	290.8	363.5	85.0	100.0	393.6
**H-20B**	140	306.5	365.9	86.8	99.9	385.7
	150	311.6	376.1	76.2	98.8	393.5
	160	305.9	367.4	85.3	99.8	390.7
	170	308.2	366.7	86.7	100.0	393.8
